# Bacteria associated with acne use glycosaminoglycans as cell adhesion receptors and promote changes in the expression of the genes involved in their biosynthesis

**DOI:** 10.1186/s12866-022-02477-2

**Published:** 2022-02-26

**Authors:** Carla Martín, Helena Ordiales, Francisco Vázquez, Marta Pevida, David Rodríguez, Jesús Merayo, Fernando Vázquez, Beatriz García, Luis M. Quirós

**Affiliations:** 1 Brill Pharma, 08022 Barcelona, Spain; 2 Instituto Universitario Fernández-Vega, Universidad de Oviedo, 33003 Oviedo, Spain; 3 Fundación para la Investigación y la Innovación Biosanitaria de Asturias (FINBA), 33011 Oviedo, Spain; 4 Departamento de Biología funcional, Universidad de Oviedo, 33006 Oviedo, Spain; 5 Servicio de Dermatología, Hospital Universitario Central de Asturias, 33011 Oviedo, Spain; 6 Departamento de Medicina, Universidad de Oviedo, 33006 Oviedo, Spain; 7 Centro Comunitario de Sangre y Tejidos del Principado de Asturias, 33006 Oviedo, Spain; 8 Departmento de Bioquímica y Biología Molecular, Universidad de Oviedo, 33006 Oviedo, Spain; 9 Servicio de Microbiología, Hospital Universitario Central de Asturias, 33011 Oviedo, Spain

**Keywords:** Acne, Bacterial infection, Glycosaminoglycan, Heparan sulfate, Chondroitin sulfate

## Abstract

**Background:**

Cell surface glycosaminoglycans (GAGs) participate in many physiological and pathological processes, including infections and inflammatory response. Acne is a common chronic inflammatory skin disorder that affects the pilosebaceous unit and has a multifactorial etiology, including bacterial colonization of the hair follicle. This study aimed to investigate the participation of GAG in the adhesion of *Propionibacterium acnes*, *Staphylococcus aureus* and *Staphylococcus epidermidis* to keratinocytes and fibroblasts of the skin by competition experiments and cell surface removal using specific liases. The alteration in the transcription of the genes responsible for the synthesis of GAG induced by the adhesion of these bacteria was also analyzed by qRT-PCR.

**Results:**

GAGs are involved in bacterial adherence to skin cells, especially fibroblasts, where chondroitin sulfate displayed the higher effect. Bacterial adherence produced different alterations in the transcription of the genes responsible for GAG structures. *P. acnes* induced mostly changes in keratinocytes, while *S. epidermidis* was the main cause of alterations in fibroblasts. These variations in gene expression affected all the stages in the biosynthesis of the main species of GAGs, heparan and chondroitin sulphate.

**Conclusions:**

GAGs species are involved in the adhesion of acne-related bacteria to skin cells in a differential manner depending on each microorganism and cellular type, although other receptors seem to exist. Bacterial adherence led to variations on gene expression in skin cells affecting GAG chains structure what, consequently, should alter their interactions with different ligands, affecting the development of acne disease.

**Supplementary Information:**

The online version contains supplementary material available at 10.1186/s12866-022-02477-2.

## Background

Human skin hosts a dense microbiota, mostly harmless or beneficial to health. Two of the principal genera are *Propionibacteria* and *Staphylococci*, predominantly distributed in sebaceous follicle and skin surface respectively. Dysbiosis of microbiota increased the susceptibility to infections and chronic inflammatory diseases, including acne [1].

Acne is a chronic disorder of the pilosebaceous unit that mainly affects the face, but that can spread to different areas and display different degrees of severity [2, 3]. Acne pathogenesis is multifactorial, resulting from excess of androgen-induced sebaceous secretion, abnormal follicle keratinization, colonization by *Propionibacterium acnes*, and inflammation process [4]. Nevertheless, recent studies have suggested that it is an inflammatory disease from the outset [5, 6].

In healthy conditions, *P. acnes* is a commensal that participates in skin maintenance; however, it acts as an opportunistic pathogen in different inflammatory diseases [7]. Diverse phylogenetic groups of *P. acnes* are differentially involved in healthy areas and acne lesions [8]. Over-colonization by this microorganism causes intensification of the inflammatory process by several different mechanisms, including enzymatic, antigenic, chemoattractant and complement activation activities [9].

Several studies have analyzed the importance of staphylococci in acne, since *Staphylococcus epidermidis* and *Staphylococcus aureus* have been found in the sebaceous follicles affected [7, 10, 11]. Moreover, interactions between *P. acnes* and different staphylococci has been largely known, with antagonist interactions to control each other proliferation by different procedures [7, 12, 13].

Due to its multifactorial etiopathogenesis, various combinations of antibiotic and anti-inflammatory therapies have been applied to control acne. Notwithstanding, improved solutions are needed to overcome urgent problems, highlighting antibiotic resistance [14].

A critical step in infections is the adhesion to human cells, which is mediated by diverse molecules, particularly glycosaminoglycans (GAGs). GAGs are linear polysaccharide chains, generally covalently bounded to specific core proteins, forming proteoglycans. They are essential in the control of multiple biological processes. GAGs are classified depending on their chemical composition in hyaluronic acid, keratan sulfate, and the two main cell surface GAGs: heparan sulfate (HS) and chondroitin sulfate/dermatan sulfate (CS/DS).

**Fig. 1:**
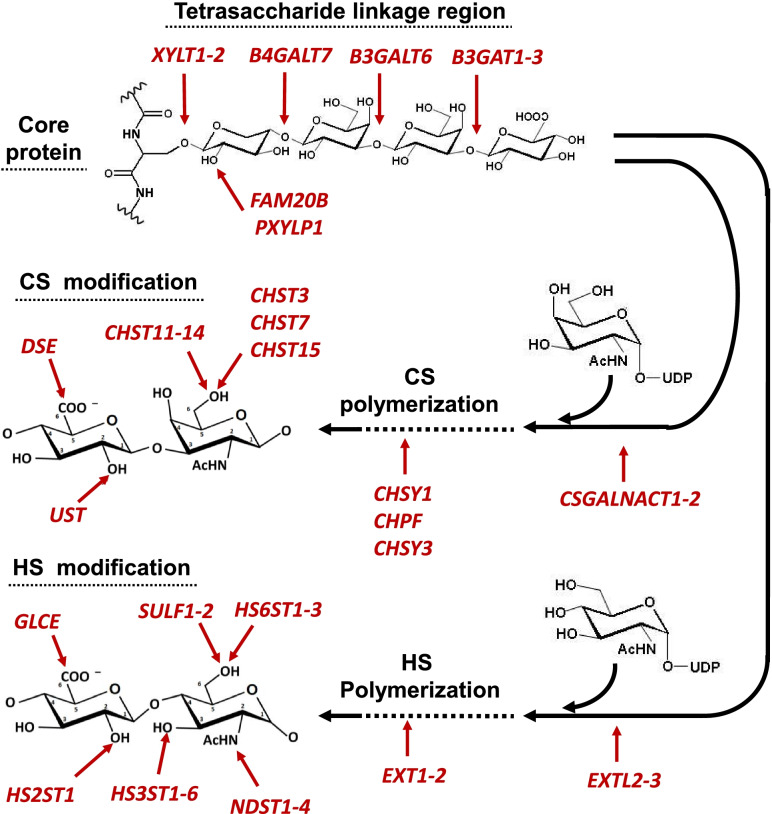
Genes involved in HS and CS biosynthesis. The different genes encoding the enzymes that catalyze the specific reactions are highlighted in red

The biosynthesis of HS and CS is complex and determine the final structure of the chains, which is dynamic and vary depending on the cell type, stage of development and physiological and pathological conditions (Fig. 1). Both GAGs share the initial steps of their biosynthesis, giving rise to the formation of a tetrasaccharide linker formed by a xylose, two galactoses and a glucuronic acid residue (GlcA). This is followed by the polymerization phase in which residues of GlcA and N-acetylglucosamine (GlcNAc) are alternately linked in the HS chains, and of GlcA and N-acetylgalactosamine (GalNAc) in those of CS [15–17]. Simultaneously to polymerization, both types of chains can be enzymatically modified at different specific positions to give rise to complex structures. In the case of CS chains, GlcA residue can be epimerizated a sulfated at C2, whereas GalNAc may be sulfated at C4 and C6 [16]. HS, for its part, can undergo a greater number of modifications according to a specific sequence that includes N-deacetylation/N-sulphation of the GlcNAc residue, epimerization and sulphation at C2 of GlcA, and sulphations in C6 and C3 of GlcNAc, resulting in a structure with alternating domains of high and little modification. Furthermore, HS can also be post-synthetically modified by extracellular sulphatases that act on C6 of specific residues [15].

CS and HS chains interact specifically with a variety of bioactive molecules [15–17], and, accordingly, participate in numerous physiological and pathological processes, including inflammatory response [18–20]. GAGs also mediate in the adherence of multiple bacteria to epithelial cells, including staphylococci, and participate in infections [21, 22]. It has been described that certain *P. acnes* strains have an adhesin that bindings to DS, and some others secrete lyases capable of degrading hyaluronic acid and CS [23–25]. Furthermore, it has been recently described that the adherence of microbiota microorganisms to epithelial cells induces important alterations in the expression of multiple genes responsible for the structure of receptor GAGs, resulting in alterations in the ability to bind to microorganisms and in other aspects of cell physiology mediated by these molecules [26].

The aim of this work is to analyze the involvement of cell surface GAGs in the adhesion of acne-related bacteria to keratinocytes and fibroblasts from human skin, as well as to determine the changes that this adhesion induces in the translation of the genes responsible for the structure of these GAGs. These data may contribute to a better understanding of the establishment of the infection, as well as the immune response associated with the disease. A better understanding of the molecular details of the disease could ultimately prove useful in the development of new therapies.

## Results

### Distinct GAG species are differentially involved in bacterial adherence to skin cells

To analyze the involvement of GAGs in bacterial adherence, cell surface GAGs were digested using specific bacterial lyases. Chondroitinase ABC and a mixture of heparinases I and III were used to degrade the CS and HS chains respectively.

**Fig. 2:**
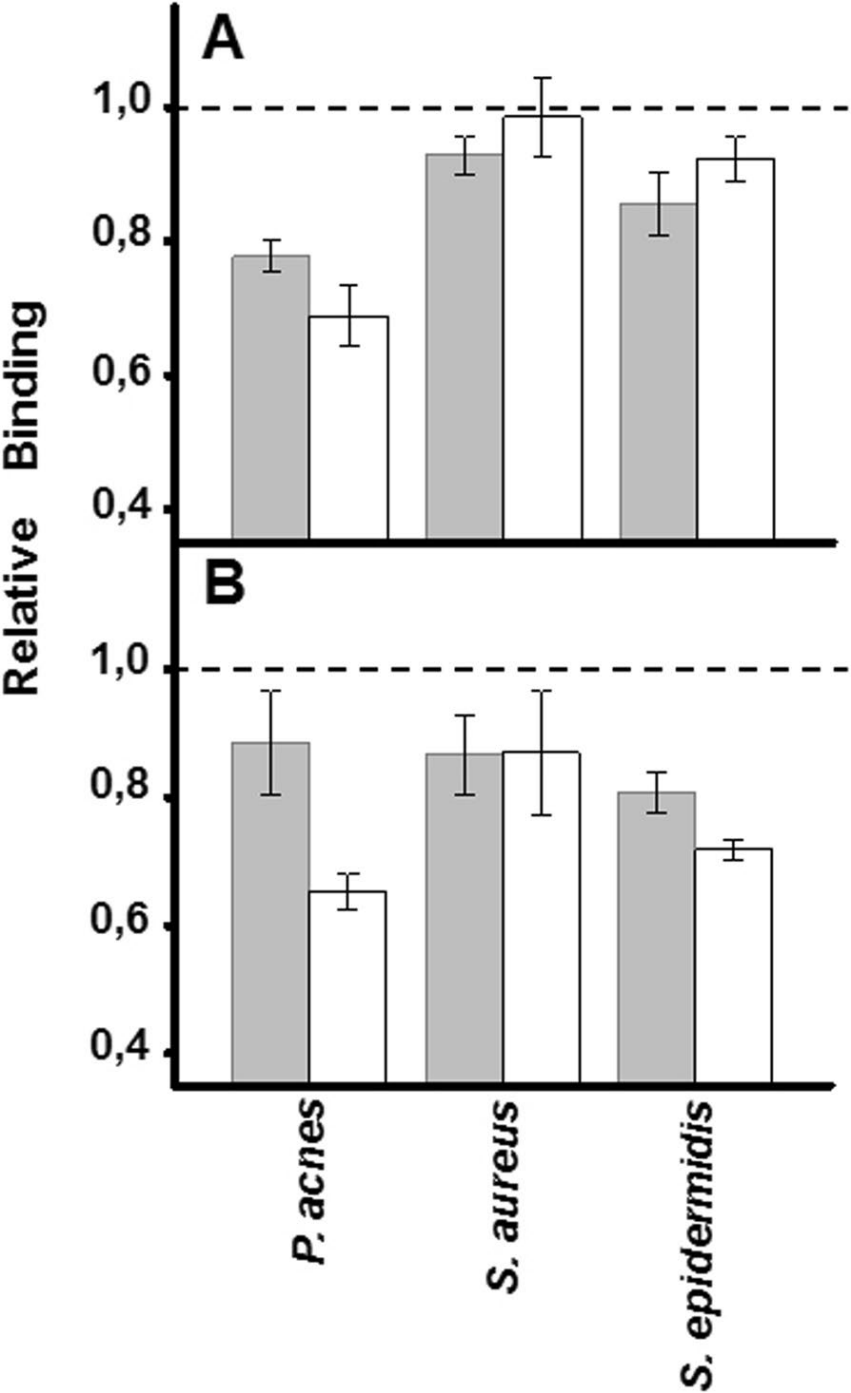
Effect of enzymatic degradation of cell GAGs on bacterial adherence to skin cells. Inhibition of bacterial attachment to (**a**) keratinocytes and (**b**) fibroblasts treated with heparinases I and III (grey bars) or chondroitinase ABC (white bars). Data were normalized using the adhesion values of bacteria to non-treated cells, which was given the arbitrary value of 1. The spreads represent the standard deviations

In keratinocytes, *P. acnes* was the microorganism whose adhesion was most affected by the digestion of GAGs. Degradation of CS chains reduced adherence by more than 30%, while removal of HS did so by around 25%. On the contrary, the effects on the adherence of staphylococci were much smaller; removal of HS decreased the binding of *S. aureus* by only about 10%, whereas treatment with chondroitinase had no effect and, in the case of *S. epidermidis*, removal of HS and CS decreased adherence by about 15 and 10%, respectively (Fig. 2A).

Regarding fibroblasts, the adherence of *P. acnes* was inhibited by around 35 and 10% by the elimination of CS and HS, respectively. Furthermore, the effects of GAG hydrolysis on staphylococcal binding were greater than those observed in keratinocytes; the degradation of both GAGs reduced the binding of *S. aureus* by similar values, around 15%, while the elimination of HS and CS reduced the binding of *S. epidermidis* by 20 and 30%, respectively (Fig. 2B).

These results suggest that cell surface GAGs are involved, at least in part, in the adherence of these bacteria. The effect is variable depending on the microorganism, the cell type and the specific species of GAG.

**Fig. 3:**
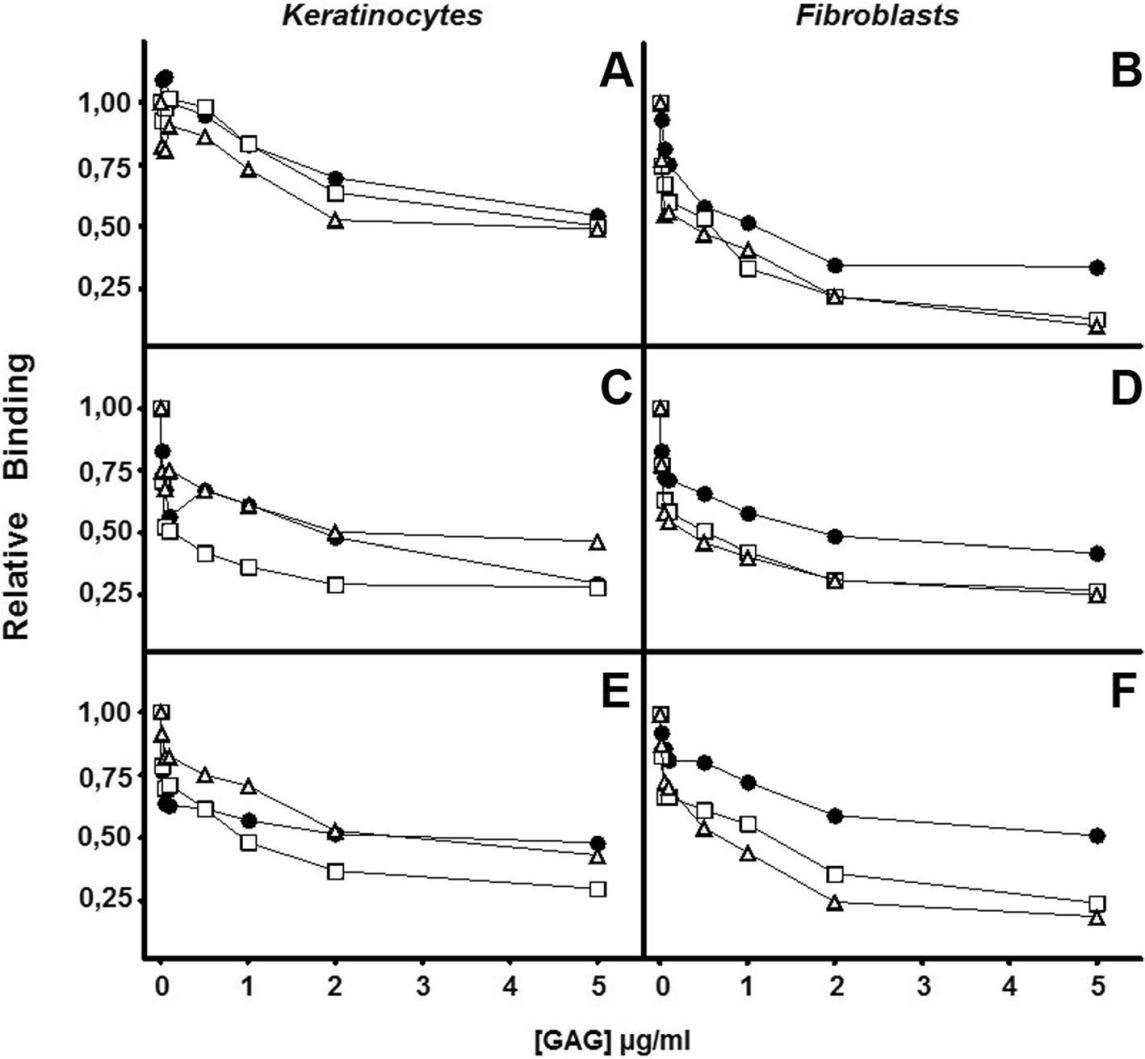
Inhibition of bacterial attachment to skin cells in the presence of different GAGs. Adhesion of *Propinebacterium acnes* (**a**, **b**), *Staphyloccocus aureus* (**c**,**d**), *Staphyloccocus epidermidis* (**e**,**f**) to keratinocytes and fibroblasts in the presence of different concentrations of HS (●), a mixture of CS (□) and a mixture of all GAGs (Δ). Data were normalized using the adhesion values of bacteria to non-treated cells, which was given the arbitrary value of 1

To analyze the participation of HS and CS chains in bacterial binding, adhesion interference experiments were carried out using commercial molecules. In all cases, the competition generated by the addition of each of the GAGs species reduced bacterial binding in a concentration-dependent manner (Fig. 3).

The addition of the saccharide chains of GAGs reduced the binding of *P. acnes* to both cell lines differently. In the case of keratinocytes, in all cases the observed inhibitory effect reached levels around 50% at the highest concentrations; however, at low concentrations the inhibitory effect of HS and CS was very similar, while the combined use of a mixture of both molecules had a greater effect (Fig. 3 A). On the other hand, in fibroblasts, both CS and the mixture of GAGs produced an identical effect, drastically reducing adherence by around 80%, while HS had a lower competitive capacity, reducing adherence by about 65% (Fig. 3 B).

With respect to staphylococci, the competition of the different species of GAGs produced important decreases in the binding of these microorganisms to both cell lines. CS reduced the adherence of *S. aureus* to keratinocytes at low concentrations, although at higher concentrations both GAGs reached similar inhibition values, around 70% (Fig. 3 C). In fibroblasts, HS showed a lesser effect, 55%, while both CS and the mixture of GAGs were able to inhibit binding around 75% (Fig. 3 D).

On the other hand, in the case of the adherence of *S. epidermidis* to keratinocytes, both HS and CS showed comparable effects at low concentrations, although at higher concentrations CS was the most interfering molecule, reaching 70%, while both HS and the mixture of GAGs only produced a 60% inhibition at high concentrations (Fig. 3E). In fibroblasts, CS showed an inhibitory effect greater than HS (75% versus 40% respectively), being comparable at high concentrations to that produced by the mixture of both molecular species (Fig. 3 F).

### Bacterial binding modifies the transcription of the genes responsible for the structure of GAGs

Interaction with bacteria can lead to changes in the structure of GAGs due to modifications in the expression of the genes that encode the enzymes involved in their biosynthesis. A transcriptomic analysis of these genes was performed in keratinocytes and fibroblasts in the absence or presence of the studied microorganisms. The study included 46 genes, 9 responsible for the synthesis of the linker that binds the saccharide chains to the corresponding core protein of proteoglycans, 14 involved in the synthesis of CS, and 21 of the synthesis of HS.

**Fig. 4:**
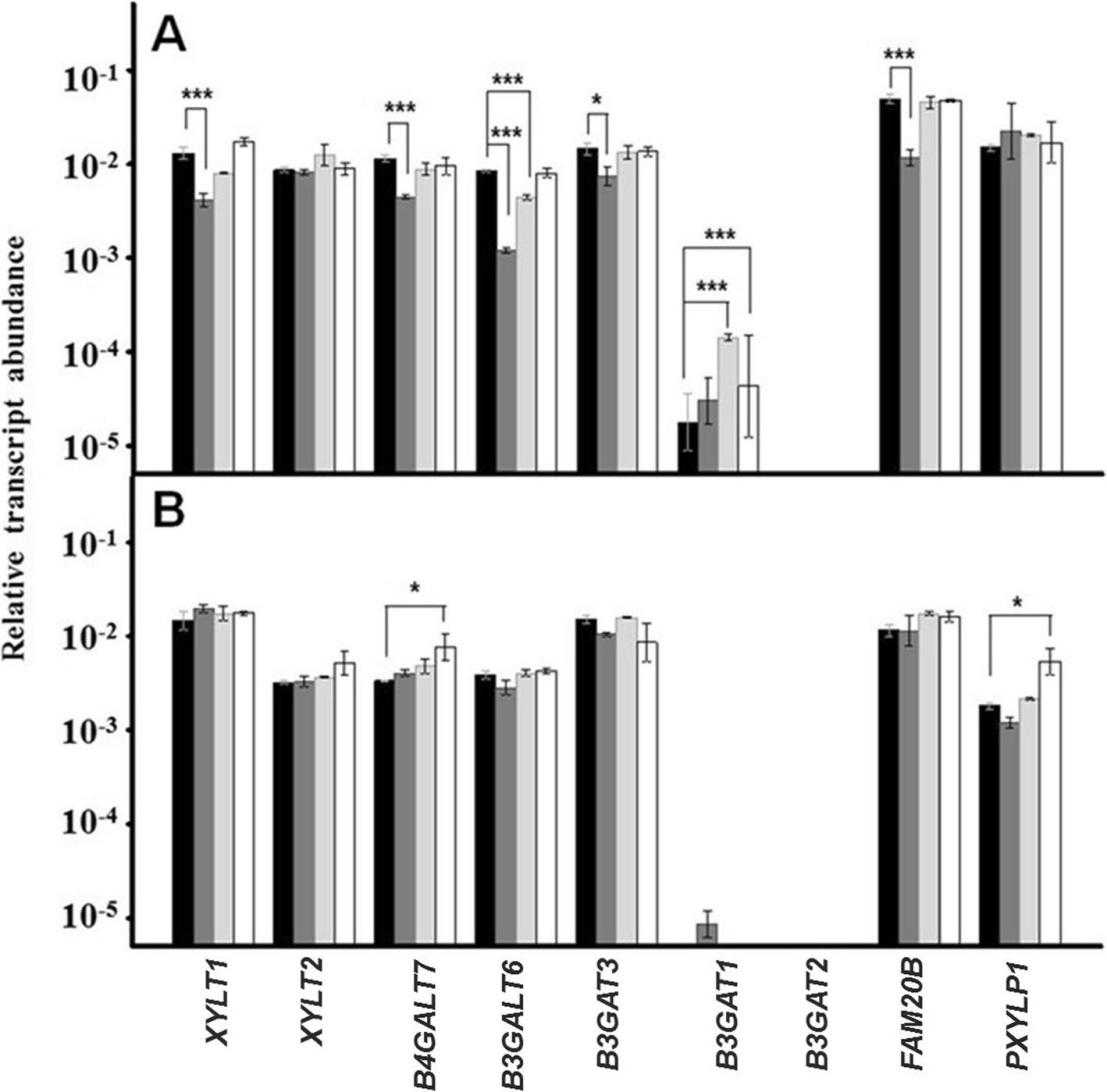
Differential transcription of genes involved in the biosynthesis of the tetrasaccharide linker. Relative transcript abundance of mRNAs for cultured keratinocytes (**a**) and fibroblasts (**b**) in the absence of bacteria (black bars), in the presence of *Propinebacterium acnes* (dark grey bars), *Staphyloccocus aureus* (light grey bars), and *Staphyloccocus epidermidis* (white bars) are plotted on a logarithmic scale. The spreads represent the standard deviations. Statistically significant differences are denoted by ***, and *, which indicate *p* < 0.001, and *p* < 0.05 respectively

In keratinocytes, the transcription of the genes involved in the synthesis of the tetrasaccharide linker was fundamentally altered by the adherence of *P. acnes*, which decreased the expression of four glycosyltransferases: *XYLT1*, *B4GALT7*, *B3GALT6* and *B3GAT3* by 70, 60, 85 and 50% respectively. *P. acnes* also down-regulated the transcription of *FAM20B*, encoding a xylose kinase, by more than 75% (Fig. 4a). In contrast, *P. acnes* did not affect the transcription of any of these genes in fibroblasts (Fig. 4b). Transcription in keratinocytes was much less affected by the binding of the two staphylococci species, with glycosyltransferase B3GAT1 being overexpressed 2.5 and 8 times by *S. epidermidis* and *S. aureus*, respectively; binding of *S. aureus* also caused about 50% down-regulation of *B3GALT6*. In the case of fibroblasts, only *S. epidermidis* altered the expression of two of the genes, *B4GALT7* and *PXYLP1*, which were overexpressed 2 and 3 times respectively (Fig. 4b).

**Fig. 5:**
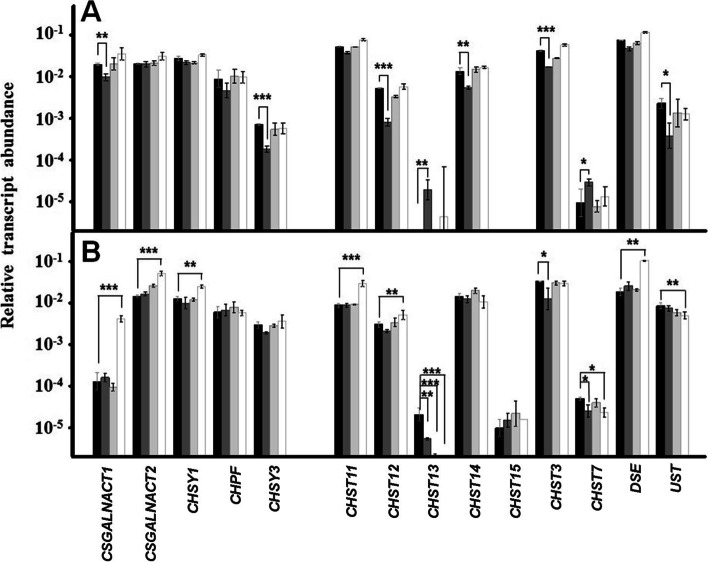
Differential transcription of genes involved in the biosynthesis of CS/DS chains in skin cells. Relative transcript abundance of mRNAs for cultured keratinocytes (**a**) and fibroblasts (**b**) in the absence of bacteria (black bars), in the presence of *Propinebacterium acnes* (dark grey bars), *Staphyloccocus aureus* (light grey bars), and *Staphyloccocus epidermidis* (white bars) are plotted on a logarithmic scale. The spreads represent the standard deviations. Statistically significant differences are denoted by ***, **, and *, which indicate *p* < 0.001, *p* < 0.01, and *p* < 0.05 respectively

CS synthesis is carried out by the enzymes encoded by 14 genes, of which 5 are involved in chain polymerization and 9 in its modification (Fig. 1). In keratinocytes, only *P. acnes* produced alterations in the transcription levels of some genes, while staphylococci did not induce any. *P. acnes* reduced the expression of the glycosyltransferases CSGALNACT1 and CHY3 by around 70%, and also affected chain sulphation at different levels. Of the genes responsible for C4 sulphation of GalNAc, *CHST12*, *CHST14* appeared downregulated 85 and 65% respectively, while certain levels of transcripts for *CHST13* could be detected, absent in the control. The C6 sulphation of GalNAc was also altered, with *CHST3* appearing decreased by 60%, while *CHST7* was overexpressed 3 times. Finally, *UST*, responsible for the C2 sulphation of the GlcA residue, was under-expressed by 80% (Fig. 5a).

When the alterations induced by bacterial adherence were analyzed in dermic fibroblasts, a very different pattern appeared from that of keratinocytes. *P. acnes* reduced the transcription of *CHST13* by 75%, and that of *CHST3* and *CHST7* by over 50%, suggesting a greater affectation of sulphation in C6, while the binding of *S. aureus* only affected the expression of *CHST13* (Fig. 5b). However, the adherence of *S. epidermidis* induced notable changes in expression, which affected more than 50% of the genes involved. Chain polymerization was affected, since the glycosyltransferases *CSGALNACT1*, *CSGALNACT2* and *CHSY1* appeared overexpressed 32, 3.5 and 2 fold respectively; *CHST11 and CHST12* were overexpressed more than 3 and 2 fold, while *CHST7* transcripts were reduced by more than 50%, all these genes being involved in the sulphation of the GalNac residue; finally, the expression of the two genes involved in the epimerization and the sulphation of GlcA was also affected, with *DSE* and *UST* being overexpressed 2 and 5 fold of respectively (Fig. 5b).

**Fig. 6:**
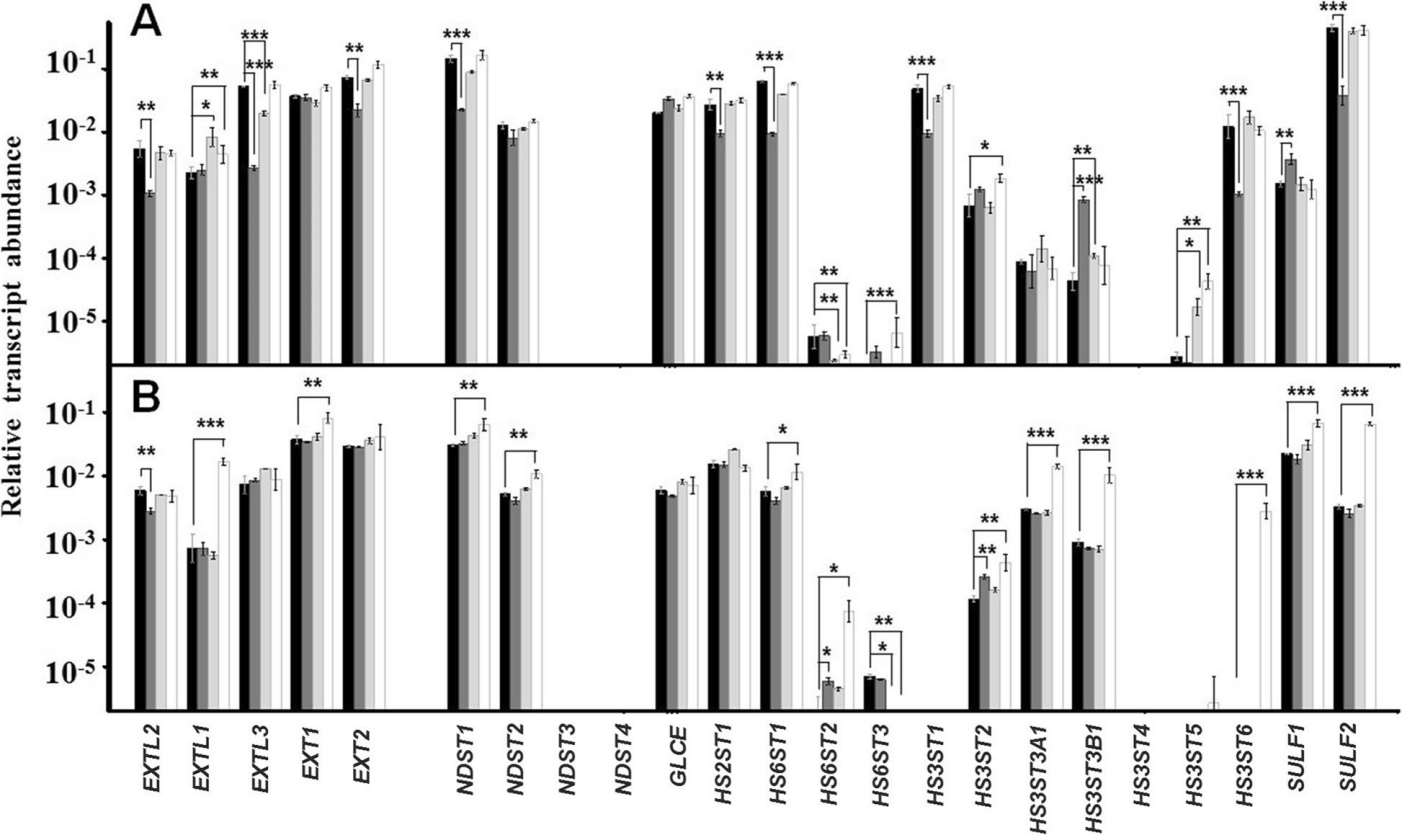
Differential transcription of genes involved in the biosynthesis of HS chains in skin cells. Relative transcript abundance of mRNAs for cultured keratinocytes (**a**) and fibroblasts (**b**) in the absence of bacteria (black bars), in the presence of *Propinebacterium acnes* (dark grey bars), *Staphyloccocus aureus* (light grey bars), and *Staphyloccocus epidermidis* (white bars) are plotted on a logarithmic scale. The spreads represent the standard deviations. Statistically significant differences are denoted by ***, **, and *, which indicate *p* < 0.001, *p* < 0.01, and *p* < 0.05 respectively

The analysis of the transcription of the genes responsible for the synthesis of HS chains in keratinocytes showed that *P. acnes* was the microorganism responsible for a greater number of alterations, affecting 11 out of the 23 genes involved. The adherence of *P. acnes* affected the enzymes responsible for the polymerization of the HS chain, reducing the expression of *EXTL2*, *EXTL3* and *EXT2* by 80, 95 and 70% respectively (Fig. 6a). The different sulphation stages of the molecule were also affected, including N-sulphation, in which *NDST1* transcripts were reduced by 85%, and C2 sulphation of GlcA, appearing *HS2ST1* deregulated by 65%. *HS6ST1* transcription decreased by about 85%, and the extracellular sulphatases *SULF1* and *SULF2* experienced an overexpression of 2.5 fold and a downregulation of more than 90% respectively, being all of these genes involved in the sulphation of C6 of the glucosamine residue. Finally, 3 of the 7 genes involved in C3 sulphation of this same residue underwent alterations, including the dysregulation of *HS3ST1* and *HS3ST6* around 80 and 90% respectively, and overexpression of *HS3ST3B1* around 20 fold (Fig. 6 a).

The binding effect of streptococci to keratinocytes was much more limited than that observed for *P. acnes*. Both microorganisms induced an overexpression of *EXTL1*, in more than 2 fold by *S. epidermidis* and around 3.5 fold by *S. aureus*, and of *HS3ST5* in 15.5 and 6 fold respectively, as well as the practical disappearance of transcripts for *HS6ST2* in both cases. Additionally, *S. aureus* also caused a diminution higher than 60% of *EXTL3* and an increase of 2.5 fold in mRNA levels of *HS3ST3B1*. On the other hand, *S. epidermidis* increased *HS6ST3* and *HS3ST2* by about 3 times (Fig. 6a).

The adherence of *P. acnes* to dermal fibroblasts produced very few alterations, limited to a 50% under-expression of *EXTL2*, and an overexpression of *HS6ST2* and *HS3ST2* by 3 and 2 fold respectively (Fig. 6b). *S. aureus* only reduced the expression to non-detectable levels of *HS6ST3*, whose expression levels in control cells were already very low compared to the *HS3ST1* isoform. However, the adherence of *S. epidermidis* altered the transcription of more than 50% of the HS biosynthetic genes, affecting almost all the stages, with the only exception of the modification of the GlcA residue. Chain polymerization was altered, resulting the glycosyltransferases *EXTL1* and *EXT1* overexpressed around 25- and more than 2-fold respectively. This bacterium also caused about 2-fold up-regulations in the expression of *NDST1* and *NDST2*, what should affect the structure of sulfated domains in the molecule. The transcriptions of the genes encoding the 3 isoforms involved in the C6 sulphation of glucosamine were affected, producing an overexpression of *HS6ST1*, the majority isoform, in more than 2 fold, as well as of *HS6ST2* in 35 fold, and the disappearance of transcripts for *HS6ST3*; furthermore, the sulphation in this residue was also affected by the overexpression of *SULF1* and *SULF2* by 3- and more than 20-fold respectively. Finally, the C3 sulphation of glucosamine was massively affected, as all the isoforms responsible for this modification detectable in fibroblasts, *HS3ST2*, *HS3STA1*, *HS3STB1* and *HS3ST6*, appeared overexpressed, the first three in 4-, 5- and 11 fold respectively, not being the isoform 6 detectable in the control (Fig. 6b).

## Discussion

The involvement of cell surface GAGs in bacterial adherence processes can be evidenced by determining the influence that their selective enzymatic degradation produces on the binding of microorganisms. The cell surface HS can be fragmented by using a mixture of heparin lyases I and III, which cleave highly sulphated and less sulphated HS domains respectively, releasing mainly disaccharides. On the other hand, for the fragmentation of chondroitin sulfate, chondroitinase ABC can be used, capable of acting on different types of CS [27].

In most cases, the reduction of the levels of GAGs by enzymatic degradation had some influence on the adherence of the microorganisms to the cells, suggesting an implication of these molecules in the union, independently of the possible existence of other cellular receptors. In keratinocytes both GAGs, but especially CS, seemed to be more relevant for *P. acnes* adhesion; both of them also seemed to be partially involved in *S. epidermidis* establishment, but only HS affected *S. aureus* adhesion. However, all GAGs exhibited a higher effect on bacterial binding to fibroblasts, particularly CS for *P. acnes* and *S. epidermidis*.

The role of the two main species of GAGs present on the cell surface, HS and CS, was analyzed by determining their ability to competitively inhibit the adherence of microorganisms as a function of the concentration of each specific species or a mixture of both. The binding of *P. acnes* to keratinocytes was inhibited in a similar way by HS and CS, the mixture of both being even more effective at low concentrations, reaching comparable levels of inhibition at high concentrations, results that suggest the implication of the two types of molecules in the binding. However, in keratinocytes, the union of the two staphylococci species showed a similar pattern, in which HS and the mixture of GAGs produced comparable effects, but in which CS had a higher inhibitory capacity, which could be reflecting more complex effects that could involve other receptors. In fibroblasts, the three microorganisms displayed similar inhibition patterns. In all cases, CS was able to interfere in the union more effectively than HS, suggesting that it is the main cell surface receptor involved. In *P. acnes* and *S. aureus* the addition of the mixture of both molecules produced inhibitions comparable to those obtained using only CS, while in *S. epidermidis* they were superior, which suggests that in this microorganism there is some cooperation between both species, as has been previously described in the case of bacterium adherence to other cell types [28].

The GAGs of keratinocytes and fibroblasts therefore showed a different role in the adherence of the tested microorganisms, which can be related to the structural variability of these molecules as a function of the cell type from which they originate. Keratinocytes are the outermost layer of the skin, becoming the main barrier of protection, while fibroblasts are located in a deeper and less-exposed layer. When microorganisms penetrate the barrier by a breach new more susceptible receptors for them are available [29]. Both layers differ in their receptors, including proteoglycans and GAGs that are differentially distributed in the skin [30].

Both GAGs are exploited by important pathogens for their adherence to different host cells [20, 21]. Subversion of GAGs by staphylococci was previously known [29], but, in this work, differential GAGs involvement in *S. epidermidis* and *S. aureus* adhesion to skin cells was found, probably due to their distinct pathogenic nature. *P. acnes* attachment was also mediated by GAGs, being CS the main mediator, agreeing with the existence of an adhesin for binding to DS [23, 24]. Although *P. acnes* have a low pathogenic and invasive potential, some strains may spread secreting hyaluronidases that degrade GAGs from extracellular matrix [25]. *P. acnes* activates several signaling pathways, leading to gene expression alterations, affecting those for cytokines and pro-inflammatory chemokines, thus it promotes inflammatory response, and is associated with diverse inflammatory diseases, as sarcoidosis or osteomyelitis [31].

GAGs present dynamic structures depending on the physiological and pathological state of the cells. Alterations in these molecules associated with the development of various pathologies, including cancer, amyloid and inflammatory diseases have been described [27, 32–34]. These molecules are also involved in the development of infectious pathologies [20], and it has been recently described that their structure is modulated as a result of the interaction with microbiota microorganisms [26]. The enormous structural diversity of GAGs seems to be regulated by the expression of the genes responsible for their synthesis [15, 16]. Although the possible existence of biosynthetic structures in which regulatory molecules could be involved has been postulated, the differential transcription of biosynthetic genes seems an essential element in the regulation of the biosynthetic process [35].

In keratinocytes, the adherence of *S. aureus* and *S. epidermidis* hardly produced alterations in the transcription levels of the genes responsible for the synthesis of HS. There was an aberrant expression of *HS6ST2* and *HS3ST5*; however, in both cases they are minor transcripts, expressed at levels around 4 orders of magnitude below the predominant isoforms for each of these reactions (*HS6ST1* and *HS3ST1* respectively), thus probably their effect on the final structure of the molecule is limited. An overexpression of *EXTL1* was also observed, although this gene encodes a GlcNAc transferase whose role is not well characterized in relation to the synthesis of HS [36]. Additionally, *S. aureus* also induced a subexpression of *EXTL3*, responsible for the initiation of elongation, although it has also been related to interactions with other molecules, which would be related to other types of biological functions [37].

In contrast to staphylococci, the adherence of *P. acnes* to keratinocytes induced changes in the transcription levels of both the genes responsible for the synthesis of HS and CS, affecting a majority of the genes whose expression could be detected in these cells: 11 of 20 for HS (55%), and 8 of 13 for CS (62%). In both cases, curiously, all the biosynthesis stages were involved except the epimerization of the GlcA residue. In both cases, they are genes with a high number of detectable transcripts, responsible for a reaction that provides the chain with conformational flexibility, facilitating the existence of specific interactions with different ligands [38]. Furthermore, these alterations may be related to those observed in the expression of the genes encoding the enzymes involved in the synthesis of the tetrasaccharide linker to the core protein. *P. acnes* induced an aberrant transcription of 5 of these genes, including at least one of the isoforms involved in each stage of polymerization (*XYLT1*, *B4GALT7*, *B3GALT6*, *B3GAT3*) and in its regulation (*FAM20B*). In contrast, staphylococci hardly produced alterations.

The changes detected in the HS biosynthesis genes affected 3 of the 5 genes responsible for chain polymerization (60%), one of the only two NDSTs expressed by these cells (*NDST1*), *HS2ST1* (encoding the only enzyme responsible for this reaction), one of the 3 6STs (*HS6ST1*), 3 of the 6 3STs (*HS3ST1*, *HS3ST3B1*, *HS3ST6*), and the two extracellular sulfatases (*SULF1*, *SULF2*). In addition, and interestingly, in all cases the species for which the alterations were detected corresponded to those that displayed a greater number of transcripts (*NDST1*, *HS6ST1*, *HS3ST1*), and consisted of subexpressions in the vast majority of cases, which suggests important changes in the final structure of the molecules, towards shorter and less sulphated chains.

Regarding the genes responsible for the synthesis of the CS chains, the adherence of *P. acnes* to keratinocytes induced an altered transcription in 2 of the 5 glycosyltransferases involved on chain polymerization (*CSGALNACT1*, *CHSY3*), 3 of the 4 sulfotransferases responsible for the modification of position 4 of GalNAc (*CHST12*, *CHST13*, *CHST14*), the 2 responsible for the sulphation of position 6 of this same residue (*CHST3*, *CHST7*), and the sulfotransferase involved in sulphation C2 of uronic acid residue (*UST*). On the other hand, and analogously to HS, the vast majority of changes consisted of subexpressions, which would suggest that the binding of *P. acnes* to keratinocytes significantly alters the structure of the CS chains, generating less sulphated chains.

When the effect of bacterial adherence to fibroblasts on the transcription of the GAGs biosynthesis genes was analyzed, the patterns obtained were radically different from those observed in keratinocytes. *P. acnes* induced overexpressions in only two genes responsible for sulphation at C6 (*HS6ST2*) and C3 (*HS3ST2*) of glucosamine. In both cases, these are genes whose number of detectable transcripts places them orders of magnitude below the majority forms, suggesting a limited impact on the structure of the molecules. In addition, *EXTL2*, encoding a glycosyltransferase of weak activity that has been related to the control of biosynthesis, was under-expressed [39].

*S. aureus* produced practically no alterations in gene transcription, limited to the under-expression of two minority forms involved in the sulphation of the HS (*HS3ST3*) and CS (*CHST13*) chains. On the contrary, *S. epidermidis* was responsible for massive alterations that affected 72% of the genes involved in the biosynthesis of HS with detectable transcription levels, and 64% of those of CS. In the case of HS, the only transcripts that did not undergo any modification were those of genes encoding enzymes modifying the uronic acid residue, including epimerization (*GLCE*), and C2-sulphation (*HS2ST1*). With a single exception, affecting to a minority isoform (*HS6ST3*), all the alterations consisted of overexpressions.

Regarding CS synthesis, the numerous transcriptional alterations observed affected genes responsible for all biosynthesis stages, including on this occasion the modifiers of the uronic acid residue (*DSE* and *UST*). In the vast majority of cases (6 out of 9), the modifications corresponded to overexpressions. Altogether, the data from dermal fibroblasts suggest that the binding of *P. epidermidis* induces notable modifications in the chains of both GAGs, apparently aimed at the production of molecules with increased sulfation patterns, in addition to modifications that affect polymerization and epimerization of the CS.

## Conclusions

The alterations described in this work reinforce previous descriptions about the ability of microorganisms to modulate the structure of the GAG chains that serve as receptors [26]. These results could also be related to previous observations that describe the alteration in the expression of enzymes involved in the synthesis of these molecules in relation to inflammatory processes [40, 41], which could also be related to the specificity of patterns observed depending on the microorganism and the cell type involved. Since the fine structure of these saccharide chains is directly related to the affinity for specific ligands, including bacterial adhesins, but also regulatory factors of inflammatory processes, these alterations seem directly related to the pathological basis of acne [6]. Further studies are needed to identify concrete consequences of changes caused by bacteria in GAGs structure and their relation to acne. Our data could rise the possibility for alternative or supplementary therapies, more efficacious against infections and over-inflammatory response in acne.

## Methods

### Skin cell lines, bacterial strains and culture conditions

Keratinocytes and fibroblasts were obtained from human biopsies at the Tissue Bank of the Community Center for Blood and Tissues of Asturias, according to Spanish law (RD 9/2017), and cells were grown as follows. Keratinocytes were cultivated with lethally irradiated 3 T3 cells in 75 cm^2^ culture flasks. The culture medium was a 3:1 mixture of Dulbecco’s modified Eagle medium (DMEM, Gibco) and Ham-F12 medium (Gibco) supplemented with 10% fetal bovine serum (FBS) (Gibco), insulin (5 mg/ml, Sigma Aldrich), hydrocortisone (0.4 mg/ml, Sigma Aldrich), triiodothyronine (1.3 ng/ml, Sigma Aldrich), cholera toxin (8 ng/ml, Sigma Aldrich) and adenine (24 mg/ml, Sigma Aldrich). Fibroblasts were cultivated in 75 cm^2^ culture flasks with DMEM supplemented with 10% FBS, penicillin (5000 IU/ml, Sigma Aldrich) and G/streptomycin (5000 μg/ml, Sigma Aldrich). In both cases, cultures were incubated at 37 °C in a humidified 5% CO_2_ atmosphere.

The *P. acnes, S. aureus* and *S. epidermidis* strain*s* used were clinical isolates obtained from the Hospital Universitario Central de Asturias. Bacteria were grown in Brain Heart Infusion (Conda) at 37 °C in a shaking incubator, except *P. acnes* which was grown in anaerobic atmosphere.

### Fluorescein labeling

Overnight bacteria cultures were washed four times with phosphate-buffered saline (PBS, Gibco), resuspended in a 0.1 mg/ml fluorescein isothiocyanate (FITC, Sigma Aldrich) solution to an A_600_ of 0.5 and then incubated in the dark at 37 °C under agitation for 1 h. After that, the excess FITC was removed by 4 washes with PBS and the pellet was resuspended in DMEM.

### Adherence assays

Bacterial adhesion to skin cell monolayers experiments were performed in 24-well plates to 70-90% confluence. The medium was removed, the cells were washed twice with PBS, and then blocked with 10% FBS in DMEM for 2 h at 37 °C in a 5% CO_2_ atmosphere. After that, cells were washed with PBS, and 100 μl of FITC-labeled bacteria in 500 μl of DMEM were added. The mixture was incubated for 1 h at 37 °C in a 5% CO_2_ atmosphere. To remove the unbound bacteria, wells were rinsed four times with 500 μl of PBS. At the end of the experiments, skin cell monolayers were disaggregated with 1% SDS and the fluorescence of the bacteria attached to them was quantified in a Perkin Elmer LS55 fluorometer, at 488 nm for excitation and 560 nm for emission. Data were normalized using the adhesion values ​​from the control, which were given a value of 1. Assays were performed at least in triplicate and the data are expressed as the mean ± SD.

### Enzymatic digestion of GAGs

Digestion of GAGs was carried out by incubation for 3 h at 37 °C in a 5% CO_2_ atmosphere with a 500 mU/ml (final concentration) mix of heparinase I and III (Sigma Aldrich) for HS, and 250 mU/ml (final concentration) of chondroitinase ABC (Sigma Aldrich) for CS. Digestion of both GAG species was achieved through successive incubations of the cell cultures with the two enzymatic mixes, washing between each with PBS. The reactions were stopped with 2 washes using PBS and the cell cultures were immediately submerged in DMEM. Next, bacterial adherence assays were carried out as describe previously.

### Adherence inhibition assays

The effect of GAGs on adherence interference was investigated by the addition of HS, CS-A, CS-B, CS-C or a mixture of all (Sigma-Aldrich) at concentrations ranging from 0.005 to 5 μg/ml to FITC-labeled bacteria before their addition to cell cultures. After this, adhesion assays were performed as indicated above.

### Gene expression modification

The influence of bacterial binding in the expression of genes for GAGs chains was tested growing cell monolayers by 70-80% confluence. After washed the cells twice with phosphate-buffered saline (Gibco), 500 μL of a bacterial solution to an A_600_ of 0.5 in 2.5 ml of DMEM was added and incubated for 1 h at 37 °C and 5% CO_2_. Unbound bacteria were removed with four washes of 500 μL of phosphate-buffered saline. Next, 2 mL of appropriate medium was added, and the culture was incubated for 16 h at 37 °C and 5% CO_2_. Finally, cells were collected for a RNA isolation.

### RNA isolation and cDNA synthesis

RNA was isolated from cultures using the RNeasy kit (Qiagen, Germany), following the manufacturer’s specifications. During purification, treatment with RNase-free DNase (Qiagen) was used to ensure the removal of contaminating residual DNA. The concentration of RNA obtained was determined spectrophotometrically by measuring absorbance using a Picodrop Microliter UV/Vis spectrophotometer (Picodrop Limited). When the samples obtained were not used immediately, they were stored at − 80 °C in aliquots until their future use.

Synthesis of cDNA was carried out using the High Capacity cDNA Transcription Kit (Applied BioSystems) following the manufacturer’s instructions. The reactions were performed in an iCycler IQ thermocycler (BioRad) using 2 μg RNA as substrate. The reaction products were cleaned using the PCR Clean-Up GenElute kit (Sigma-Aldrich) following the manufacturer’s recommendations. Finally, the aliquots containing the cDNA were diluted 1:20 with water and stored at − 20 °C until use.

### qRT-PCR reactions

All qRT-PCR reactions were carried out at least four times in a final volume of 10 μl. The reactions involved 1 μl of the diluted cDNA as template, 2 μl of primer pair mix (200 nM final concentration) and 5 μl of SYBR Green PCR Master Mix (Applied Biosystems), and they were assembled in 96-well microtiter plates. Plates were sealed with optical film and centrifuged at 2500 rpm for 5 min before being placed into a Real-Time ABI Prism Detection System (Applied Biosystems; Foster City, CA) using the following cycling conditions: 50 °C for 2 min, 95 °C for 10 min, and 40 cycles of 95 °C for 15 s and 60 °C for 60 s. Following the thermal cycling and data collection steps, amplimer products were analyzed using a melt curve program (95 °C for 1 min, 55 °C for 1 min, then increasing 0.5 °C per cycle for 80 cycles of 10 s each). For each amplification the presence of a single peak with a Tm corresponding to that previously calculated was verified. In those cases where the amplifications were not adequate, new primer pairs were designed. Actin was included on each plate as a control gene to compare run variation and to normalize individual gene expression. The sequences of the primers used for each gene are described in supplementary Table 1.

### Statistical analysis

All analyses were performed using the program Statistica (Statsoft Inc.; Tulsa, OK). Mean values were compared between two samples by the Mann-Whitney *U*. *P* < 0.05 was accepted as significant.

## Supplementary Information

**Additional file 1: Table S1.** qRT-PCR primer sequences.

## Data Availability

All data generated or analysed during this study are included in this published article.

## Abbreviations

CS: Chondroitin sulfate
DS: Dermatan sulfate
GAGs: Glycosaminoglycans
GalNAc: N- acetylgalactosamine
GlcA: Glucuronic acid
GlcNAc: N- acetylglucosamine
HS: Heparan sulfate
