# Supplementary material for: Bacteria associated with acne use glycosaminoglycans as cell adhesion receptors and promote changes in the expression of the genes involved in their biosynthesis
Source: BMC Microbiol. 2022 Feb 26;22:65. doi: 10.1186/s12866-022-02477-2 (PMC8881830; doi:10.1186/s12866-022-02477-2)
Supplement: Supplementary file 1 — Additional file 1: Table S1. qRT-PCR primer sequences. [file 12866_2022_2477_MOESM1_ESM.pdf]

Additional file 1

Table S1: qRT-PCR primer sequences

Gene	Gene ID	Primer sequence
XYLT1	6382	F 5'- CTCAGGTGCAGGTGCTTTG<br>R 5'- CTGCGTGTCCTTCCAAGTG
XYLT2	6383	F 5'- GATGACGATGACTACGCTTCTG<br>R 5'- TGGAAGTGGTCGAGATGTTG
B4GALT7	9672	F 5'- CTCCTTTCCCGATGATGAAC<br>R 5'- CGACTCCTGCTCGAAGTAGC
B3GALT6	6385	F 5'- GGCAGGAATCTGATGACTTTG<br>R 5'- TCTAGAGGCACCAAGGGATG
B3GAT3	2817	F 5'- CATCGGGTGTGGAGAGTG<br>R 5'- TGAGCGTGTCCCTGTTGTC
B3GAT1	221914	F 5'- CTGGGACACGACCTGGAC<br>R 5'- GCCATCCAGTCATCTGCATAC
B3GAT2	2719	F 5'- CTGCTTCAGTCTGCAAGTATGG<br>R 5'- GTGGAGTCAGGCTTGGGTAG
FAM20B	2239	F 5'- AGTGTGGTCAGCGAACAGTG<br>R 5'- CAAACATATCATTCAGGGATTTCTC
PXYLP1	2262	F 5'- GCCGCCCTGTAAGAACAC<br>R 5'- TCATTCCATGCTTCTCTTTGC
CSGALNACT1	55790	F 5'- TCAGGGAGATGTGCATTGAG<br>R 5'- AGTTGGCAGCTTTGGAAGTG
CSGALNACT2	55454	F 5'- GCCATTGTTTATGCCAACCA<br>R 5'- ATCCACCAATGGTCAGGAAA
CHSY1	22856	F 5'- GCCCAGAAATACCTGCAGAC<br>R 5'- GCACTACTGGAATTGGTACAGATG
CHPF	79586	F 5'- GGTGCACTATAGCCATCTGGA<br>R 5'- GGCACTTCGGAAATGAGG
CHSY3	337876	F 5'- GACTCAGTGTGTCTGGTCTTACG<br>R 5'- TTGCTATTGTGAAGGTCTTGGA
CHST11	50515	F 5'- CGCTGCTGGAAGTGATGA<br>R 5'- AGGATAAAGGATCCCAAGCAA
CHST12	55501	F 5'- GTAGCCGACAAATCCTTCCA<br>R 5'- ACCGGTTTACCTCTGACTTGAC
CHST13	166012	F 5'- CCGGCATTTGGAAACAGA<br>R 5'- TCCAGGTCATAGAGCTTCTGC
CHST14	113189	F 5'- CCACTGCCTAATGTCACCAA<br>R 5'- ATGACAGGCAGAAGCACAGA
CHST15	51363	F 5'- GTGCCAGGAATAAAGTTCAACA<br>R 5'- CACTGGATAAGTCCCGAGTGA
CHST3	9469	F 5'- TGCACAGCCTGAAGATGAGA<br>R 5'- CAGCTTGTCTGAGACCCTTGA
CHST7	56548	F 5'- GATCCGGGTCAGTCACCA<br>R 5'- GACAGATTGCCCCCACAG
DSE	29940	F 5'- GTCCAGAGGCACTTCAACATC<br>R 5'- AGTCCGCAATAGCCACAGTC

UST	10090	F 5'- ACCATGGACCACCTCCTAGTAA<br>R 5'- CACACTTGCCTACCCTGTTGTA
EXTL2	2135	F 5'- TGAACTGGAAACCAATGCAG<br>R 5'- AGGAAATTGCTGCCAAACTG
EXTL1	2134	F 5'- GATGAGAGGCTCCCACTTCA<br>R 5'- CCTCCAGAGTGGTATGGATGA
EXTL3	2137	F 5'- CTCCGCCATGACGAAATC<br>R 5'- AGTTGGAGTTGTAGAGCCAGGA
EXT1	2131	F 5'- GAGACAATGATGGGACAGACTTC<br>R 5'- CTCTGTCGCTGGGCAAAG
EXT2	2132	F 5'- CTGGGACCATGAGATGAATA<br>R 5'- GATATCCCCAGGCATTTTGTA
NDST1	3340	F 5'- CTGCCCTCTACCTGTTCCTG<br>R 5'- AACTGGATCTCCTCAAAGGTCTC
NDST2	8509	F 5'- CAAGAGCTGCGTACCAACC<br>R 5'- GAGGGTCCGTGTGTAGTTCAG
NDST3	9348	F 5'- CCTTGCAGAAGAGATGTTTGG<br>R 5'- GTAGCAGGATCAGTTCTTAGTTGTTG
NDST4	64579	F 5'- GACATTGGGCTCCATCTGAC<br>R 5'- GCTGCTGTCCATCAATAATTAGC
GLCE	26035	F 5'- TGTGGAAGTCCGAGACAGAG<br>R 5'- CTGGATTGGATAGAAATAGCCTTG
HS2ST1	9653	F 5'- TGGAGATGATTATAGACCAGGGTTAC<br>R 5'- GCTATGGCCACAGAAGAACG
HS6ST1	9394	F 5'- GCAGGGAGTGGAGCTAACAG<br>R 5'- AACAGTTCCAGTTCCCGAAA
HS6ST2	90161	F 5'- CGGTGCGATCTTCTCCAA<br>R 5'- AGGACGATCACGGCAAATAG
HS6ST3	266722	F 5'- CAACCACAGCCACACCAG<br>R 5'- CTTCTTCCATCACACATATGAAGAG
HS3ST1	9957	F 5'- CAGCCAGATGCCCTTCTC<br>R 5'- AGACTCGCTCAGGCACTTTG
HS3ST2	9956	F 5'- GATTGGTACAGGAGCCTGATG<br>R 5'- GGAGCCTCTTGAGTGACAAAG
HS3ST3 A1	9955	F 5'- GGCCGAGAGAACCTGAACTC<br>R 5'- CGAGCGACAGTGACTTCCA
HS3ST3 B1	9953	F 5'- GCAGATCTTGCCTCGATGTC<br>R 5'- GCGCACGAGTACAGGAACATA
HS3ST4	9951	F 5'- TAGAGCCGCACTTCTTCGAC<br>R 5'- GGTTATTTGCCCATCCAAAG
HS3ST5	222537	F 5'- CATCCGGCAGTAGTCAAAGC<br>R 5'- TTGTGATTTGCTGAGGGTAGG
HS3ST6	64711	F 5'- GCCCTGCTGGAGTTTCTG<br>R 5'- GCGCTCGTAGCACCTGTC
SULF1	23213	F 5'- CCAGCAGAAGCCAAAGAAAG<br>R 5'- GAACGTGTCTGCCGAGTATG
SULF2	55959	F 5'- GCCTGCAAGAGAAGGACAAG<br>R 5'- AGCAGCTTGCGGAGTTTC
